# Different transcriptome profiles between human retinoblastoma Y79 cells and an etoposide-resistant subline reveal a chemoresistance mechanism

**DOI:** 10.1186/s12886-020-01348-6

**Published:** 2020-03-06

**Authors:** Wen-ping Song, Si Zheng, Hong-juan Yao, Xiao-fei Zhou, Rui Li, Cheng-yue Zhang, Jun-yang Zhao, Lie-wei Wang, Rong-guang Shao, Liang Li

**Affiliations:** 1 Department of Pharmacy, Affiliated Cancer Hospital of Zhengzhou University, Henan Cancer Hospital, No.127 Dongming Road, Zhengzhou, 450008 China; 2 Key Laboratory of Antibiotic Bioengineering of National Health and Family Planning Commission (NHFPC), Institute of Medicinal Biotechnology (IMB), Chinese Academy of Medical Sciences and Peking Union Medical College (CAMS & PUMC), NO.1 Tiantan Xili, Beijing, 100050 China; 3 Institute of Medical Information (IMI) & Library, Chinese Academy of Medical Sciences and Peking Union Medical College (CAMS & PUMC), NO.3 Yabao Road, Beijing, 100020 China; 4 Department of Ophthalmology, Beijing Children’s Hospital, Capital Medical University, NO. 56 Nanlishi Road, Beijing, 100045 China; 5 Division of Clinical Pharmacology, Department of Molecular Pharmocology and Experimental Therapeutics, Mayo Clinic, Rochester, MN 55905 USA

**Keywords:** Retinoblastoma, Transcriptome profile, RNA sequencing (RNAseq), Differentially expressed genes (DEGs), Chemoresistance, Y79/EDR resistant subline

## Abstract

**Background:**

Retinoblastoma (RB) is the most frequent pediatric retinal tumor. In the present study, to elucidate chemoresistance mechanisms and identify potential biomarkers in RB, we utilized RNA sequencing (RNAseq) technological platforms to reveal transcriptome profiles and identify any differentially expressed genes (DEGs) between an etoposide drug-resistant subline (Y79/EDR) and parental Y79 cells.

**Methods:**

To test whether Y79/EDR cells showed resistance to antineoplastic agents for RB, we treated the cells with etoposide, carboplatin and vincristine and analyzed them with a Cell Counting Kit-8 (CCK-8). Y79/EDR and parental Y79 cells were used for RNAseq and bioinformatics analysis to enable a genome-wide review of DEGs between the two lines using the DESeq R package (1.10.1). Then, DEG enrichment in Kyoto Encyclopedia of Genes and Genomes (KEGG) pathways was analyzed with KOBAS software. Next, real-time quantitative reverse transcription polymerase chain reaction (real time QRT-PCR) and cytotoxicity assays were performed to experimentally and functionally validate the identified candidate biomarkers.

**Results:**

Y79/EDR cells showed resistance to etoposide, carboplatin and vincristine at different concentrations. In total, 524 transcripts were differentially expressed in Y79/EDR cells based on analysis of fragments per kilobase of transcript per million fragments mapped (FPKM); among these, 57 genes were downregulated and 467 genes were upregulated in Y79/EDR cells compared to parental Y79 cells. We selected candidate DEGs, including *ARHGAP9*, *HIST1H4H*, *RELN*, *DDIT4*, *HK2*, *STC1* and *PFKFB4,* for mRNA expression validation with real time QRT-PCR assays and found that the expression levels determined by real time QRT-PCR were consistent with the RNAseq data. Further studies involving downregulation of *ARHGAP9* with a specific siRNA showed that *ARHGAP9* altered the cellular sensitivity of Y79 cells to etoposide and carboplatin.

**Conclusion:**

Our initial findings provided a genomic view of the transcription profiles of etoposide-induced acquired resistance in RB. Follow-up studies indicated that *ARHGAP9* might be a chemoresistance biomarker in RB, providing insight into potential therapeutic targets for overcoming acquired chemoresistance in RB. These findings can aid in understanding and overcoming chemoresistance during treatment of RB in the clinic.

## Background

Retinoblastoma (RB) is the most frequent pediatric retinal tumor and is initiated by biallelic inactivation of *RB1*, the first discovered tumor suppressor gene [1]. This primary intraocular pediatric cancer is gaining increasing attention from researchers with approximately 8000 new cases diagnosed every year, particularly in Asia and Africa [2, 3]. Children diagnosed early with RB are commonly treated with photocoagulation, cryotherapy, or irradiation and tumor enucleation [4, 5]. Survival of children with RB is markedly dependent on an early diagnosis shortly after the detection of symptom [5, 6].

However, in contrast to the excellent survival rates of malignant childhood RB in Europe and the United states, that in China is very low; children diagnosed with RB in China are always in the advanced D or E stage, and the survival rate is only 26% [5]. Additional chemotherapy before or after enucleation is necessary to prevent cases of hematogenous spreading or involvement of the central nervous system [5, 7]. Recurrence and metastasis of high-risk advanced RB is still a major obstacle for successful therapy in China [5, 8].

Chemotherapy resistance to antineoplastic agents, including carboplatin, etoposide and vincristine, is a major problem in the treatment of high-risk advanced RB [5, 8, 9]. Etoposide is well known for its use in the treatment of many malignancies, such as Hodgkin’s disease, lung cancer, ovarian cancer, myeloma, gastric cancer and breast cancer [10–13]; the drug is a DNA topoisomerase-II inhibitor that can directly bind to DNA and cause DNA damage [14]. Previously, we successfully generated an etoposide drug-resistant subline (Y79/EDR) from the parental human retinoblastoma cell line Y79 and explored potential mechanisms related to etoposide resistance. Preliminary results indicated that Y79/EDR cells showed significant resistance to etoposide mediated by the PI3K/AKT and p53 signaling pathways, which promoted cell proliferation and apoptosis inhibition [15].

In the present study, to elucidate the chemoresistance mechanism and identify potential biomarkers in RB, we generated transcriptome profiles of Y79/EDR and parental Y79 cells and distinguished any candidate differentially expressed genes (DEGs) between the two lines before performing functional and technical validation studies.

## Methods

### Cell cultures and treatments

The human retinoblastoma cell line Y79 was obtained from the Cancer Hospital of the Chinese Academy of Medical Sciences and was cultured in RPMI-1640 medium (Thermo Fisher Scientific, Waltham, MA, USA) with 20% (v/v) fetal bovine serum (Thermo Fisher Scientific, Waltham, MA, USA), penicillin G (100 U/mL) and streptomycin (100 μg/mL) under a humidified atmosphere of 5% CO_2_ at 37 °C. Y79/EDR cells were maintained by treating parental Y79 human retinoblastoma cells with a tolerance concentration of etoposide (Sigma-Aldrich, MO, USA) as previously described [15].

### Detection of drug resistance

Cell Counting Kit-8 (CCK-8) (Dojindo, Kumamoto, Japan) was used to detect drug resistance. Y79/EDR and parental Y79 cells were seeded at a density of 2.0 × 10^4^ cells per well with 200 μL of medium in 96-well plates and treated with different concentrations of etoposide (Sigma-Aldrich, MO, USA), carboplatin (Sigma-Aldrich, MO, USA) and vincristine (Sigma-Aldrich, MO, USA) for 48 h, respectively. Cells treated with phosphate-buffered saline (PBS) served as the controls. Then, 20 μL of CCK-8 solution was added to each well and incubated for 4 h at 37 °C. The absorbance was measured at 450 nm using a microplate reader (BioTek, Vermont, USA). GraphPad Prism 5 was used to plot the drug concentration-cell survival curves. SigmaPlot 10.0 was used to calculate IC_50_, and resistance index (RI) was calculated according to IC_50_ as the following formula:

$$ \mathrm{RI}={\mathrm{IC}}_{50}\ \left(\mathrm{Y}79/\mathrm{EDR}\right)/{\mathrm{IC}}_{50}\ \left(\mathrm{Y}79\right) $$

### RNA extraction

Total RNA was extracted from Y79/EDR and parental cells with TRIzol (Thermo Fisher Scientific, Waltham, MA, USA) according to the manufacturer’s protocol and purified with a NucleoSpin RNA Clean-up kit (Macherey-Nagel, NucleoSpin®, Germany). RNA degradation and contamination were assessed on 1% agarose gels. RNA purity and concentrations were detected using a NanoPhotometer spectrophotometer (Implen, CA, USA) and a Qubit RNA Assay Kit in a Qubit 2.0 Fluorometer (Thermo Fisher Scientific, Waltham, MA, USA), respectively. RNA integrity was assessed using the RNA 6000 Nano Kit of the Agilent Bioanalyzer 2100 system (Agilent Technologies, Santa Clara, CA, USA).

### Library construction for RNA sequencing

A total of 1 μg of RNA per sample was used as input material for RNA sample preparation. All procedures for cDNA library construction were performed using an NEBNext UltraTM RNA Library Prep Kit for Illumina (NEB, Ipswich, MA, USA) following the manufacturer’s recommendations. Sequencing of the libraries was carried out on an Illumina HiSeq 2500 platform, and paired-end reads were generated (raw data).

### Sequencing data analysis

Quality control of the raw data was performed, including removal of reads containing adapter and poly-N sequences and removal of low-quality reads, to obtain clean data. In addition, the quality score and GC content of the clean reads were calculated. Clean reads with a perfect match or only one mismatch were mapped to the Genome Reference Consortium assembly GRCh37 using TopHat2 [16] for further analysis and annotation. Quantification of gene expression levels was estimated based on fragments per kilobase of transcript per million fragments mapped (FPKM) with the following formula: FPKM = cDNA fragments/mapped fragments (millions) × transcript length (kb). We performed RNAseq analysis on the platform BMKCloud (www.biocloud.net).

### Determination and clustering analysis of DEGs

Prior to differential gene expression analysis, for each sequenced library, the read counts were adjusted with the edgeR program package through one scaling normalized factor. Differential expression analysis of two samples was performed using the DESeq (2010) R package. *P* values were adjusted using q values [17]. Fold change (FC) ≥ 2 and q value < 0.005 were set as the threshold for significant DEGs.

### Pathway enrichment analysis

Gene Ontology (GO) enrichment analysis of DEGs was executed with the R package GOseq based on Wallenius’ noncentral hypergeometric distribution [18], which can adjust for gene length bias in DEGs. Terms with KS value < 0.05 (Kolmogorov–Smirnov) were considered significantly enriched. The Kyoto Encyclopedia of Genes and Genomes [19] (KEGG, http://www.genome.jp/kegg/) was used to predict the enriched pathways of the DEGs. KOBAS software was used to test the statistical enrichment of DEGs in KEGG pathways [20].

### Real-time quantitative reverse transcription polymerase chain reaction (real time QRT-PCR) validation

To validate the expression levels of the DEGs obtained from RNAseq, we selected 7 genes for real time QRT-PCR analysis. Total RNA from parental Y79 and Y79/EDR cells was isolated with TRIzol reagent (Thermo Fisher Scientific, Waltham, MA, USA), and then cDNA was synthesized with a Transcriptor First Strand cDNA Synthesis Kit (Roche, Mannheim, Germany). Real time QRT-PCR was conducted with a SYBR® Premix Ex Taq™ II kit (TaKaRa, Kusatsu, Japan). All procedures were performed according to the manufacturer’s instructions. The 2^-ΔΔCt^ method was used to determine relative expression levels with the GAPDH gene as an internal control [21]. To further calculate log_2_ FC (fold change) between Y79/EDR and Y79 cell lines from real time QRT-PCR, an equation of log_2_ [2^-ΔΔCt^ (Y79/EDR)/ 2^-ΔΔCt^ (Y79)] was used to compare with that from RNAseq. The primers used are listed in Additional file 1: Table S1.

### Transfection and RNA interference of selected genes

To explore the relationship between the 7 identified genes and etoposide resistance, we knocked down these genes in parental Y79 cells to determine their effects on drug sensitivity. Three short interfering RNA (siRNA) sequences targeting different regions of each gene were transiently transfected at a concentration of 100 nM into parental Y79 cells with Lipofectamine RNAiMAX (Thermo Fisher Scientific, Waltham, MA, USA). The siRNA sequences are listed in Additional file 2: Table S2. Scrambled siRNA was used as a negative control (RiboBio, Guangzhou China). Then, some of the cells were collected 6–8 h after transfection and seeded into 96-well plates for drug sensitivity analysis, while others were harvested for real time QRT-PCR after 48 h.

### Cytotoxicity assay

Parental Y79 cells transfected with siRNA of 7 candidate genes were seeded in 96-well plates, respectively, and treated with different concentrations of etoposide, carboplatin and vincristine for 48 h. Then, CCK-8 was used to analyse changes of drug sensitivity after knockdown of candidate genes.

To observe drug-induced changes in *ARHGAP9* mRNA expression, parental Y79 cells were seeded at a density of 5.0 × 10^5^cells per well with 2 mL of medium into 6-well plates and treated with IC_50_ of carboplatin and etoposide for 24 h, following with detection of *ARHGAP9* mRNA expression by real time QRT-PCR.

### Statistical analysis

Statistical analyses of the transcriptome profiles were described in above subsections, respectively. The rest analyses were conducted using Student’s t-test for functional validation studies. The quantitative data were presented as mean ± SEM.

## Results

### Multiple generations of etoposide resistance in the Y79/EDR human RB subline

**Fig. 1:**
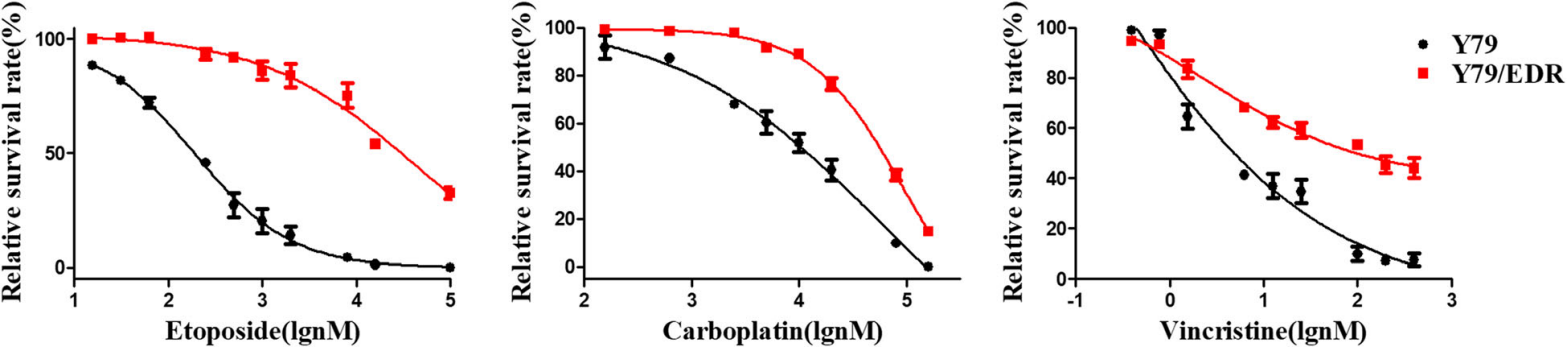
Y79/EDR cells showed significant resistance to etoposide, carboplatin and vincristine compared to parental Y79 cells. Cytotoxicity was detected by CCK-8 assays. Data are expressed as mean ± SEM

Y79/EDR cells were maintained successfully for constitutive treatment with 1 μM etoposide [15]. To test if Y79/EDR cells showed resistance to other antineoplastic agents for RB, we treated the cells with etoposide, carboplatin and vincristine which are widely used for retinoblastoma therapy. As shown in Fig. 1, Y79/EDR cells showed significant resistance not only to etoposide, but also to carboplatin and vincristine, as compared to parental Y79 cells. The resistance index (RI) of Y79/EDR cells to etoposide, carboplatin and vincristine reached 148.36, 5.21 and 24.61, respectively, as shown in Table 1, according to the previous criteria [22]. It indicated that the established Y79/EDR cells showed multidrug resistance (MDR). This finding showed that different transcriptome profiles could be involved and that DEGs needed to be identified to elucidate the intrinsic mechanism of chemoresistance during RB treatment.

**Table 1:** Comparison of drug sensitivity between Y79/EDR and Y79 cells

Drug	IC50 (nmol/L, mean ± SEM)		RI
	Y79	Y79/EDR	
Etoposide	195.84 ± 16.44	28,609.55 ± 24.0211	148.36
Carboplatin	10,755.82 ± 1694.46	56,022.36 ± 3639.34	5.21
Vincristine	5.08 ± 0.74	125.02 ± 32.60	24.61

*RI* Resistance index.

### RNAseq data and determination of DEGs

**Fig. 2:**
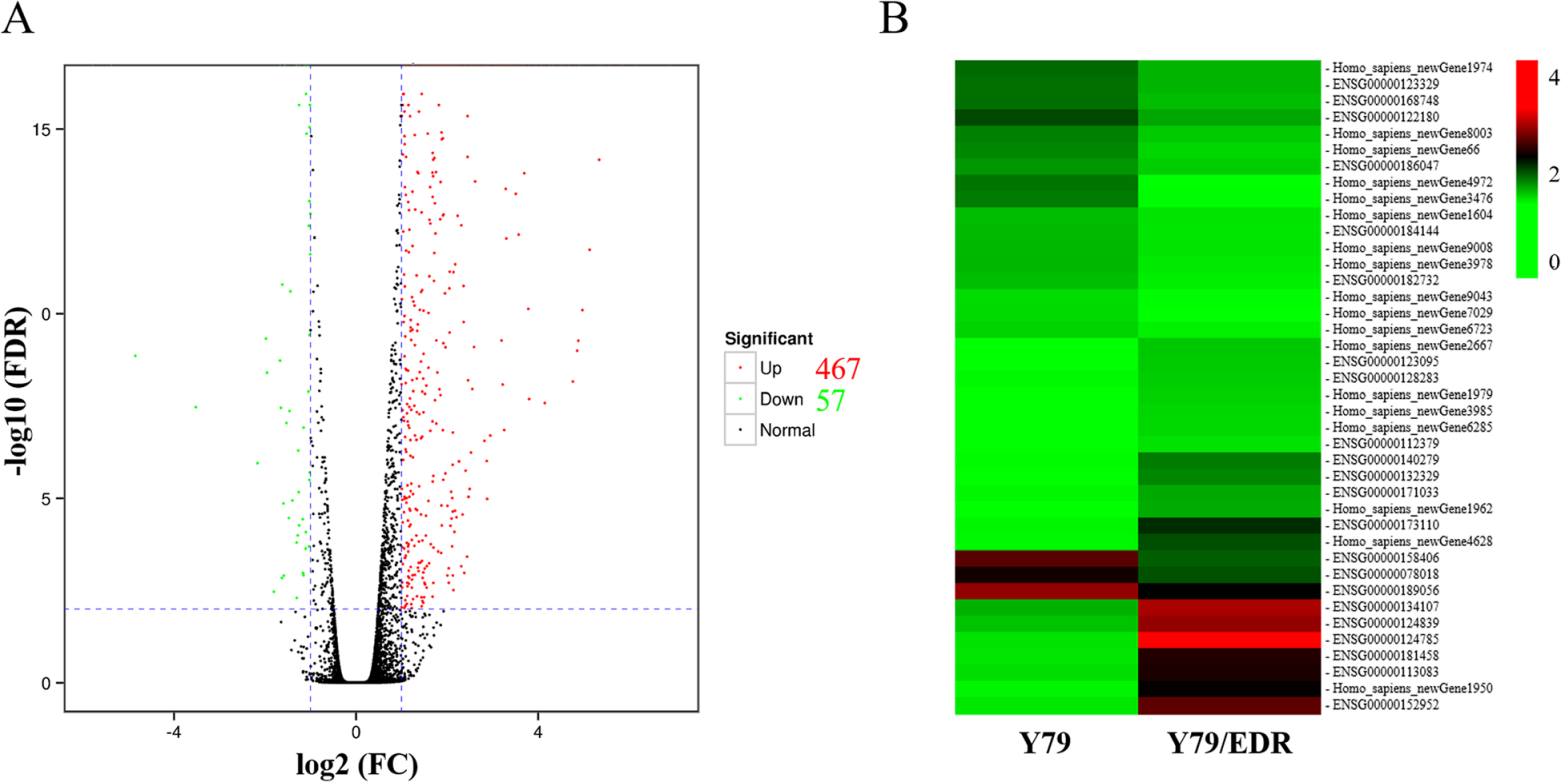
DEGs between parental Y79 and Y79/EDR cells. **a** Volcano plot of DEGs. FDR: false discovery rate, FC: fold change. The green and red dots indicated down and up-regulated genes, respectively. The black dots expressed genes without significantly differential expression. **b** Heatmap of 20 downregulated and 20 upregulated genes with top log_2_FC. Color indicated expression level of DEGs with log_2_ (FPKM+ 1)

In the present study, two cDNA libraries from Y79 and Y79/EDR cells were established and successfully sequenced. After quality filtering, a total of 245.7 million high-quality clean single-end reads were generated to yield RNAseq data for the two samples. Most of the genes in the Y79 and Y79/EDR cDNA libraries, representing 82.46 and 83.08% of the reads, respectively, were mapped to the reference genome as shown in Table 2. In total, 524 transcripts were differentially expressed with an FC ≥ 2 and a false discovery rate (FDR) < 0.01 in Y79/EDR cells in FPKM analysis. Among those genes, 57 were downregulated and 467 were upregulated in Y79/EDR cells compared to parental Y79 cells (Fig. 2a and Additional file 3: Table S3). Figure 2b showed 20 downregulated and 20 upregulated genes with top log_2_FC, which were highlighted in bold in Additional file 3: Table S3.

**Table 2:** The data from RNA sequencing analysis of Y79 and Y79/EDR

Sample ID	Clean reads	Mapped reads	Unique mapped reads	Multiple mapped reads	GC content (%)	% ≥ Q30 (%)
Y79	135,821,250	112,001,603 (82.46%)	103,505,931 (76.21%)	8,495,672 (6.26%)	53.32	95.77
Y79/EDR	109,849,280	91,258,763 (83.08%)	88,208,898 (80.30%)	3,049,865 (2.78%)	52.94	95.72

### GO and KEGG analysis of the DEGs

**Fig. 3:**
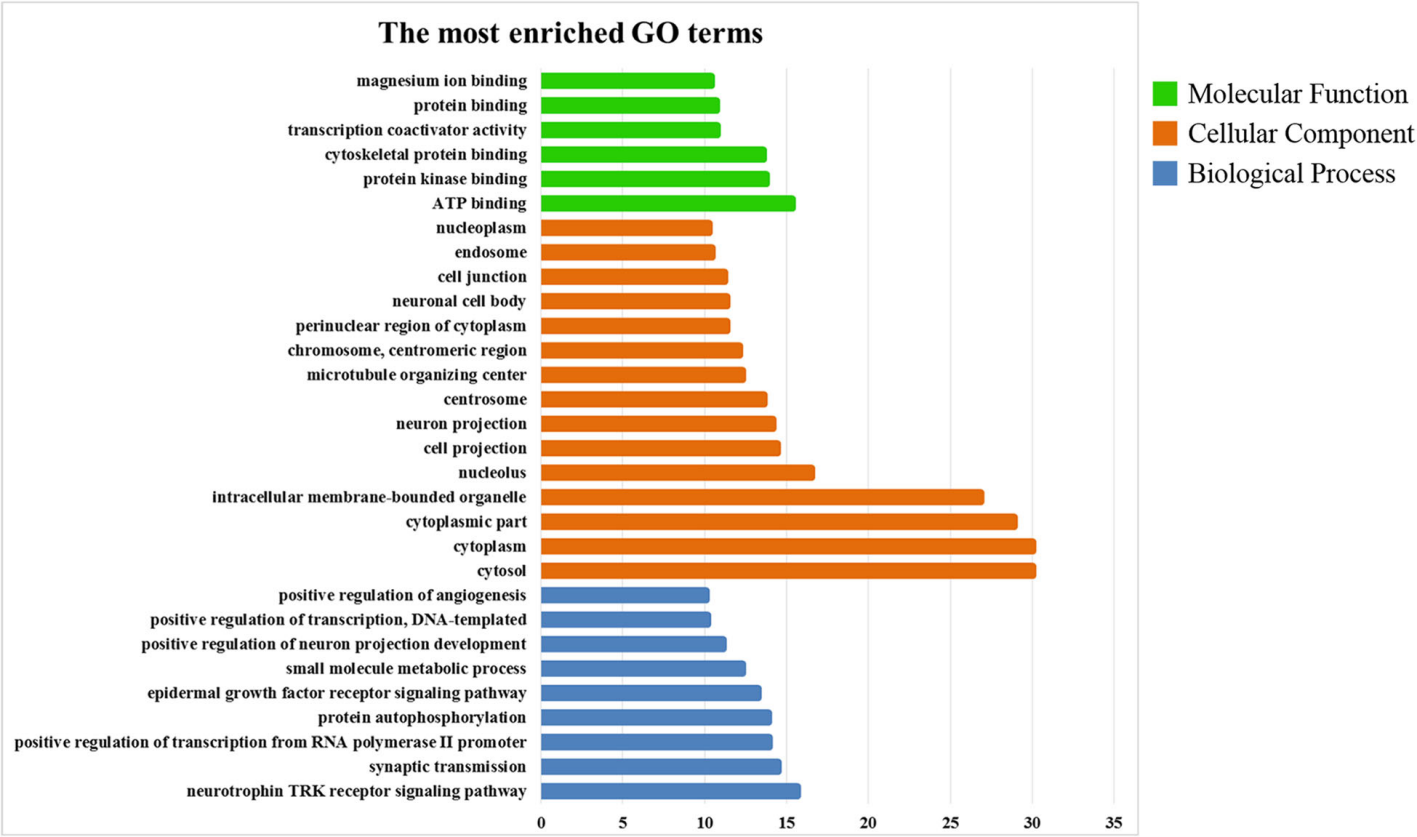
The 30 most enriched GO terms of DEGs between parental Y79 and Y79/EDR cells. The terms of molecular function, cellular component and biological process were marked as green, orange and blue bars, respectively

**Fig. 4:**
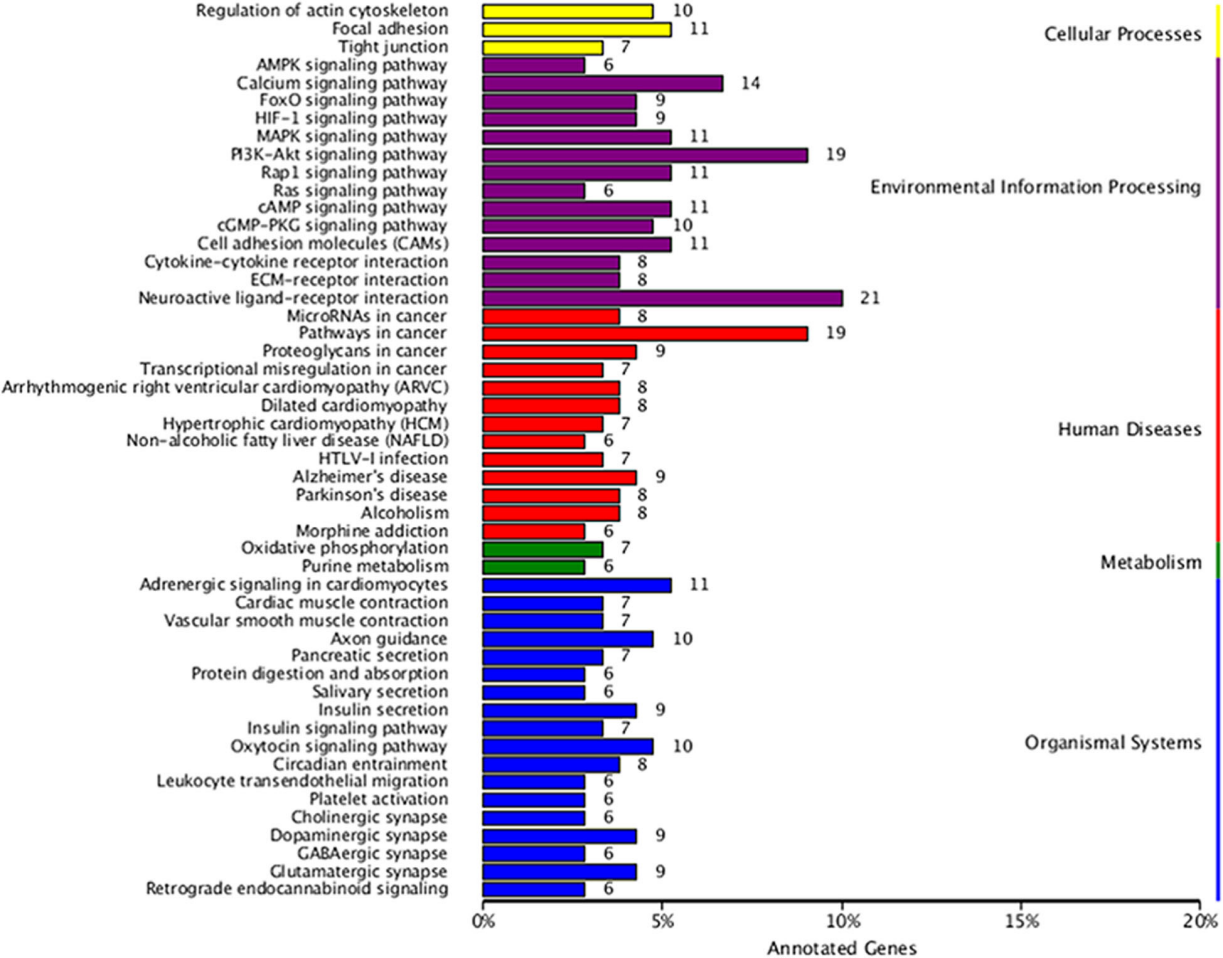
The top 50 pathways of KEGG pathway enrichment analysis of DEGs. X-axis: percentage of DEGs in the same pathway, Y-axis: functional pathways

GO, which contains three main ontologies: biological process, cellular component and molecular function, was used to analyze the obtained DEGs. The top 30 most enriched GO terms are summarized in Fig. 3. In the category of molecular function, the ‘ATP binding’, ‘protein binding’ and ‘transcription coactivator activity’ terms were the most enriched, while the most abundant cellular component terms were ‘cytosol’, cytoplasm’, ‘nucleolus’ and ‘cell junction’. The biological processes of the DEGs were related to the ‘neurotrophin TRK receptor signaling pathway’, the ‘EGFR signaling pathway’ and ‘positive regulation of neuron projection development and angiogenesis’. The results of GO enrichment analysis of the DEGs are shown in Additional file 4: Table S4. To determine whether genes associated with etoposide resistance were involved in specific pathways, the KEGG database was used to annotate the pathways of the DEGs. The results revealed that the 524 DEGs were annotated with 148 pathways, as shown in Additional file 5: Table S5. Figure 4 shows the top 50 enriched pathways, among which the most significant were related to the actin cytoskeleton, focal adhesion and tight junctions, AMPK signaling, calcium signaling, PI3K-AKT signaling, pathways in cancer, arrhythmogenic right ventricular cardiomyopathy (ARVC), neuroactive ligand-receptor interactions, FoxO signaling, and p53 signaling pathways. Our previous study suggested that resistance mechanisms in Y79/EDR cells might be related to promotion of cellular proliferation and inhibition of cell apoptosis mediated by the AKT signaling pathway [15]. The changes in the PI3K-AKT signaling pathway indicated by GO enrichment and KEGG analysis further confirmed these previous results, demonstrating the reliability of our technical platform and research strategy.

### Experimental real time QRT-PCR validation for RNAseq data

**Fig. 5:**
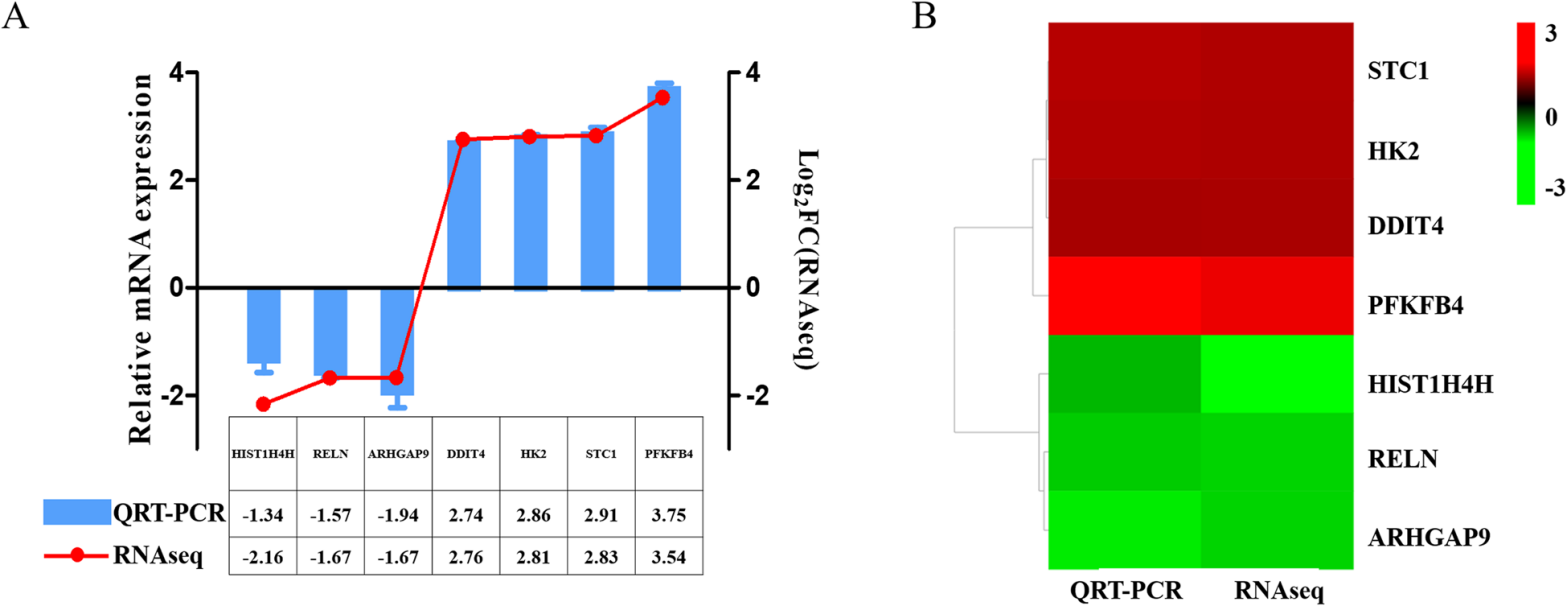
Validation of DEGs by real time QRT-PCR. **a** Relative expression levels of DEGs from real time QRT-PCR compared with RNAseq. Blue bars: real time QRT-PCR, red line: RNAseq, Y-axis (left): log_2_ FC (fold change) calculated by the equation of log_2_[2^-ΔΔCt^(Y79/EDR)/ 2^-ΔΔCt^(Y79)](real time QRT-PCR), Y-axis (right): log_2_FC(RNAseq). **b** Heatmap of the expression patterns of the 7 selected DEGs obtained from real time QRT-PCR and RNAseq. Color indicated expression levels of DEGs

To quantitatively validate the results from the Y79/EDR and Y79 transcriptome data, we conducted real time QRT-PCR evaluation of 7 candidate DEGs, including *ARHGAP9*, *HIST1H4H*, *RELN*, *DDIT4*, *HK2*, *STC1* and *PFKFB4* (Table 3), and determined the expression profiles of these selected genes. These 7 genes were selected from among the DEGs based on two criteria: (1) they were top downregulated or top upregulated genes determined by FC, and (2) the FPKM of Y79 wasn’t below 1.5. We found that the expression levels of the 7 genes determined by real time QRT-PCR were generally in good agreement with the RNAseq data, as shown in Fig. 5. These results indicated that gene expression patterns determined by real time QRT-PCR were consistent with those determined by RNAseq analysis, supporting the accuracy of our transcriptome data.

**Table 3:** The 7 genes selected from DEGs with criteria

Gene ID	Gene symbol	Y79_FPKM	Y79/EDR_FPKM	FDR	log_2_FC	Alteration by Y79
ENSG00000158406	*HIST1H4H*	8.60	1.90	1.11E-06	−2.16	Down
ENSG00000189056	*RELN*	13.04	4.27	0	−1.67	Down
ENSG00000123329	*ARHGAP9*	1.55	0.63	1.88E-09	−1.67	Down
ENSG00000168209	*DDIT4*	51.26	346.84	0	2.76	Up
ENSG00000159399	*HK2*	22.64	161.80	0	2.81	Up
ENSG00000159167	*STC1*	2.74	18.93	0	2.83	Up
ENSG00000114268	*PFKFB4*	1.72	19.31	0	3.54	Up

### Functional validation of candidates

**Fig. 6:**
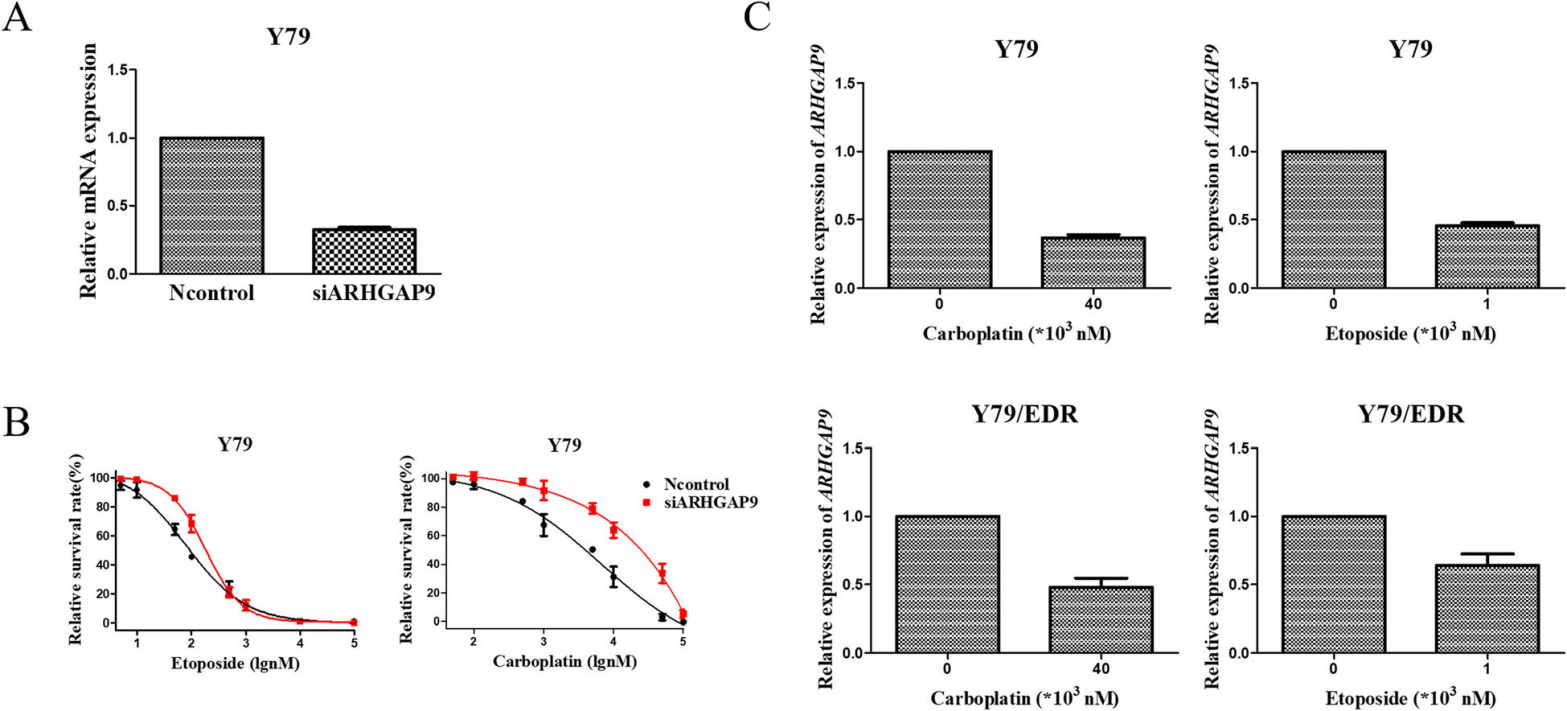
*ARHGAP9* is associated with the sensitivities of parental Y79 cells to carboplatin and etoposide. **a** Knockdown of ARHGAP9 in parental Y79 cells with siRNA detected by real time QRT-PCR. **b** Cellular sensitivities of parental Y79 cells to etoposide and carboplatin after downregulation of ARHGAP9 by CCK-8 assays. Data are expressed as mean ± SEM. **c** Relative expression levels of *ARHGAP9* in parental Y79 and Y79/EDR cells after treated with carboplatin and etoposide. Data are expressed as mean ± SEM. *P < 0.05, **P < 0.01

To determine the functional impact of the above candidate genes on drug responses in RB cells, we used specific siRNA pools to knock down the 7 selected candidate genes, followed by real time QRT-PCR and CCK-8 assays for etoposide, carboplatin and vincristine. Our studies showed that downregulation of *ARHGAP9* with a specific siRNA significantly increased the cellular resistance of Y79 cells to etoposide and carboplatin, as shown in Fig. 6a and b. However, there are no significant changes in drug sensitivity after knockdown of the other six candidate genes. Furthermore, etoposide and carboplatin significantly decreased the mRNA expression of *ARHGAP9* (Fig. 6c). Our confirmatory experiments thus revealed that *ARHGAP9* might be involved in the molecular mechanisms underlying etoposide-induced chemoresistance.

## Discussion

In the present study, RNAseq technology and gene expression profile analysis revealed that etoposide significantly altered the transcriptomic profile of Y79/EDR cells. GO and KEGG pathway enrichment analyses identified several key pathways involved in etoposide-induced acquired resistance. These pathways were linked to cellular processes and environmental information processing involving regulation of the actin cytoskeleton, focal adhesion and tight junctions, AMPK signaling, calcium signaling, ARVC, FoxO signaling, PI3K-AKT signaling and cancer pathways, providing us with insights into potential new molecular therapeutic targets in RB treatment. The results were also demonstrated in our previous report, which indicated that Y79/EDR cells showed significant resistance to etoposide that was mediated via the PI3K/AKT and p53 signaling pathways [15]. Thus, we have successfully experimentally confirmed our study design and hypothesis.

As well known, acquired drug resistance appears to be a relatively common issue throughout the administration of treatment, leading to cancer treatment failure and a patient relapse. As evolving of cancer cells during drug treatment, it is possible that a variety of related molecules in cancer cells can be altered by aberrant microenvironment due to genomic instability or intratumor heterogeneity, leading to MDR to cancer therapy [23]. For instance, levels of reactive oxidative species (ROS) in cancer cells were modulated during chemotherapy, further resulting in MDR regulated by various associated molecules including NADPH oxidases (NOXs), thioredoxin reductases (TrxRs) and nuclear factor erythroid 2-related factor 2 (Nrf2), and so on [24, 25]. Other molecules such as P-gp, multidrug resistance protein 1 (MRP1) and BCRP have also been demonstrated to be involved [26]. Moreover, it suggests that resistance to therapy may occur through multiple somatic events simultaneously within the same tumor [27]. For example [28], following BRAF inhibitor therapy in BRAF V600 mutant melanoma, individual tumors were found to develop multiple resistance events, including NRAS and MEK1 mutations in one patient and two distinct NRAS mutations in another. It was not surprising that Y79/EDR cells showed significant resistance not only to etoposide, but also to carboplatin and vincristine, suggesting some molecules altered in genetic status or expression. Accumulating evidence shows that the expression of markers related to stemness is crucial for tumor maintenance and the mediators of resistance. Polygenic resistance mechanisms might contribute to multiple chemoresistance [27]. Our finding with regarding to MDR in Y79/EDR cells encouraged us to elucidate intrinsic mechanism of MDR in Y79/EDR cell line. Therefore, we performed RNA sequencing to discover any MDR-associated genes and identify potential prognostic markers or therapeutic targets for RB treatment. That would be important for clinical significance and targeted drug development to overcome acquired chemoresistance.

The *ARHGAP9* gene encodes a member of the Rho-GAP family of GTPase-activating proteins that has substantial GAP activity towards several Rho-family GTPases in vitro, converting them to an inactive GDP-bound state. ARHGAP9 has been implicated in regulating adhesion of hematopoietic cells to the extracellular matrix, which is correlated with cell proliferation, migration and invasion in several cancers [29–35]. A previous report indicated that ARHGAP9 expression in breast cancer was correlated with poor patient survival, implying that *ARHGAP9* could act as an oncogene. Their findings revealed that silencing *ARHGAP9* inhibited proliferation, migration, invasion and induced cell cycle G0/G1 arrest and apoptosis in breast cancer cells, effects that might be mediated by significant inhibition of ERK and p38 activity [34, 35]. Sun et al. also demonstrated that *ARHGAP9* knockdown suppressed gastric cancer cell proliferation, migration and invasion [36].

Different from the above findings, another study indicated that *ARHGAP9* expression was significantly lower in hepatocellular carcinoma (HCC) tissues than that in normal liver tissues, and the overall survival time of patients with lower *ARHGAP9* expression was significantly shorter than those with its higher expression. Further experimental overexpression of *ARHGAP9* significantly inhibited cell proliferation, migration and invasion in HCC, as well as lung metastases in vivo [33]. Furthermore, Takefuji et al. found that the mRNA level of *ARHGAP9* was strongly detected in hematopoietic cells, which inhibited cell migration, spreading and adhesion [32]. Recent results from a bioinformatics analysis of patients with breast cancer and healthy individuals revealed that high levels of *ARHGAP9* expression were associated with better prognosis, including preferable relapse-free survival and overall survival. They concluded that *ARHGAP9* might be a promising target for precision treatment of breast cancer [37]. In another report, weighted gene co-expression network analysis (WGCNA) was used to predict the intrinsic relationship or correlation between gene expression in head and neck squamous cell carcinoma (HNSCC). *ARHGAP9* were identified to be used as a biomarker and therapeutic target of HNSCC [38]. These above findings suggested that ARHGAP9 had controversial effects on cell proliferation, migration and invasion among those different cancer types. This inconsistence might be attributed to difference in action mechanisms of ARHGAP9 in various cancers where genetic instability, cellular response and formation of the tumor microenvironment might also differ owing to tissue-derived specificities and heterogeneity. Certainly, more research and clinical studies are required to explore ARHGAP9 functions in different tumors. In addition, we had also noticed that the levels of *ARHGAP9* mRNA expression in Y79/EDR cells were significantly lower than that in the parental Y79 cells. Therefore, to determine if ARHGAP9 may play an important role in RB progression and chemoresistance, we next selected *ARHGAP9* as the candidate gene and validated its effect on drug resistance experimentally.

In our study, we first observed that *ARHGAP9* downregulation significantly altered cellular susceptibility to antineoplastic agents (Fig. 6a, b) and that etoposide and carboplatin decreased the mRNA levels of *ARHGAP9* (Fig. 6c). These results imply that *ARHGAP9* may be a diagnostic and prognostic marker or a potential new molecular therapeutic target. We speculate that its functions differ mainly due to cell type specificity and different development stages of tumor progression, although more clinical data are needed to draw definitive conclusions. Furthermore, our transcriptome profiling and enrichment analysis results for Y79/EDR cells showed that the top ranked DEGs were involved in regulation of the actin cytoskeleton, focal adhesion and tight junctions. Such findings imply that the *ARHGAP9* gene might also play a very important role in those functions in cell proliferation, migration and invasion of RB tumors and in chemoresistance, similar to the suggestions of previous reports [36].

According to recent reports in China, the failure of adjuvant therapy in most patients post enucleation is mainly due to chemoresistance, leading to bottle-necks in treatment for high-risk advanced RB. In a retrospective study to observe the treatment and prognosis for different degrees of invasion of eye tissue in RB and identify the indications for post-enucleation adjuvant therapy, 537 children were recruited who had been diagnosed with unilateral RB and had received enucleation from January 2006 to December 2012 in Beijing Tongren Hospital which is the Top one rank of Ophthalmic hospitals in China, and were divided them into three groups according to their number of histopathologic high-risk factors including invasion of the optic nerve posterior to the ethmoid plate and extensive invasions of the choroid, sclera, anterior chamber, iris, and ciliary body. Chemotherapy was not recommended for patients with no risk factors following enucleation, while patients with at least one risk factor received corresponding treatment. As a result, of the 537 RB patients who received enucleation, 28 had recurrences (5.2%), and 25 died (4.7%), resulting in an overall survival rate of 95.3%. On the contrary, of the 168 with histopathologic risk factors, 26 had recurrences, and 24 died, suggesting much higher irresponsive frequency of chemotherapy at 29.8% (50 patients) [39]. A total of 202 unilateral RB patients post enucleation without prior treatment were enrolled in a retrospective study from Zhongshan Ophthalmic Center as one of the best famous Ophthalmic hospitals in China between January 2003 and February 2011, followed with or without adjuvant therapy. The locations of tumor invasion, treatment and survival condition of patients were recorded. In the end of study, the 5-PEFS (5 year probability event-free survival) in total was 88.6% (179 cases) with 23 patients died of relapse or invasion and metastasis, whereas the 5-PEFS of the patients with transection line of optic nerve involvement were only 40.0%.Among those, the 5-PEFS of patients accepted treatment was significantly higher than that of untreated ones (54.5% versus 0%), however, the irresponsive frequency of chemotherapy (45.5%) was still high for high-risk advanced RB even with treatment mainly due to drug resistance [40]. Another similar retrospective study in the same hospital between January 1997 and December 2001 showed that the 5-PEFS of a total of 102 RB patients recruited post enucleation was 79.41% (81 cases) and 20.59% (21 cases). However, the 5-PEFS of the patients with transection line of optic nerve involvement was only 14.29% in Log-rank analysis including 7 cases, among which 6 having adjuvant chemotherapy died mostly because of chemoresistance with 85.7% of the frequency of drug resistance [41].

Our confirmatory experiments validated our RNAseq results (Fig. 5). Further experimental studies are warranted to verify the results of the present study. We realize that the limitation of this study is that the transcriptomic data were obtained from only one resistant subline of Y79 cells. It is more convincible to create etoposide-resistant cell models and interpret drug resistance for RB therapy by using more than one cell line. In fact, we initially selected two common human RB cell lines (Y79 and WERI-Rb1) to prepare for etoposide-resistant cell lines, however, we found that WERI-Rb1 cell susceptibility to etoposide is much lower than that of Y79. Few WERI-Rb1 cells remained alive after treated even with 1 nM of etoposide, which didn’t allow us to create its resistant cell model. Therefore, we only used Y79 cells and successfully constructed an Y79/EDR cell line previously for the follow-up studies. More biologically representative RB cell lines, as well as a large cohort of patient samples, would be needed to generate genomic, transcriptomic, epigenomic and proteomic profiles for the complex molecular signatures of RB. Interestingly, in another ongoing study, we have recruited advanced high-risk RB patients post enucleation and chemotherapy to collect peripheral blood samples from both cured and relapse/or metastasis patients. After determined patient prognosis, we compared whole exome sequencing (WES) data between those two groups of patients followed with validation by using more clinical samples. The result from the association analysis indicated that one SNP in *ARHGAP9* was significantly associated with good prognosis (*P* < 0.01), consistent with the observation from WES data. The above results from clinical validation further convinced us to conduct research on *ARHGAP9* identified from the transcriptomic data using the in vitro resistant cell line model in the present study, confirming that *ARHGAP9* may indeed play an important role in RB chemoresistance [42]. Clearly, more in vitro and in vivo functional studies should be performed to elucidate the etoposide-induced chemoresistance mechanism in advanced RB. The use of multiple drug-resistant sublines or models will be necessary to deepen understanding of the mechanisms of drug-induced cytotoxicity and acquired resistance. Finally, deep sequencing of genetic loci involved in *ARHGAP9* mutations and amplifications will be needed to identify more predictive biomarkers for diagnosis and treatment in the future. Further mechanistic studies will be helpful in determining the functions of *ARHGAP9* in RB cell proliferation, migration, invasion and metastasis, as well as chemoresistance.

## Conclusions

Our initial findings provided a genomic view of the transcriptional profiles of etoposide-induced acquired resistance in RB. Follow-up studies indicated that *ARHGAP9* might be a chemoresistance biomarker of RB, providing insight into potential therapeutic targets for overcoming acquired chemoresistance in RB. These findings can aid in understanding and overcoming chemoresistance during treatment of RB in the clinic.

## Supplementary information

**Additional file 1: Table S1.** Primers of selected DEGs for real time QRT-PCR.

**Additional file 2: Table S2.** The target sequence for short interfering RNA.

**Additional file 3: Table S3.** A total of 57 downregulated and 467 upregulated genes in Y79/EDR as compared to parental Y79 cell line. Note: Bold, 20 downregulated and 20 upregulated genes with top log_2_FC.

**Additional file 4: Table S4.** Details of DEGs obtained by GO enrichment analysis (biological process, cellular component, molecular function). Note: KS, Kolmogorov–Smirnov.

**Additional file 5: Table S5.** The enriched pathways of KEGG analysis.

## Data Availability

The datasets used during the current study are available from the corresponding author on reasonable request.

## Abbreviations

5-PEFS: 5 year probability event-free survival
ARVC: Arrhythmogenic right ventricular cardiomyopathy
CCK-8: Cell Counting Kit-8
DEGs: Differentially expressed genes
FDR: False discovery rate
FPKM: Fragments per kilobase of transcript per million fragments mapped
GO: Gene Ontology
HCC: Hepatocellular carcinoma
HNSCC: Head and neck squamous cell carcinoma
KEGG: Kyoto Encyclopedia of Genes and Genomes
PBS: Phosphate-buffered saline
RB: Retinoblastoma
Real time QRT-PCR: Real-time quantitative reverse transcription polymerase chain reaction
RI: Resistance index
RNAseq: RNA sequencing
WES: Whole exome sequencing
WGCNA: Weighted gene co-expression network analysis
Y79/EDR: Y79 etoposide drug-resistant subline

## References

[1] Friend SH, Bernards R, Rogelj S, Weinberg RA, Rapaport JM, Albert DM (1986). A human DNA segment with properties of the gene that predisposes to retinoblastoma and osteosarcoma. Nature..

[2] Dimaras H, Corson TW, Cobrinik D, White A, Zhao J, Munier FL (2015). Retinoblastoma. Nat Rev Dis Primers.

[3] Dimaras H, Kimani K, Dimba EAO, Gronsdahl P, White A, Chan HSL (2012). Retinoblastoma. Lancet.

[4] Shields CL, Shields JA (2010). Retinoblastoma management: advances in enucleation, intravenous chemoreduction, and intra-arterial chemotherapy. Curr Opin Ophthalmol.

[5] Zhao JY, Li SF, Shi JT, Wang NL (2011). Clinical presentation and group classification of newly diagnosed intraocular retinoblastoma in China. Br J Ophthalmol.

[6] AlAli A, Kletke S, Gallie B, Lam WC (2018). Retinoblastoma for pediatric ophthalmologists. Asia Pac J Ophthalmol (Phila).

[7] Yanik O, Gunduz K, Yavuz K, Tacyildiz N, Unal E (2015). Chemotherapy in retinoblastoma: current approaches. Turk J Ophthalmol.

[8] Jain M, Rojanaporn D, Chawla B, Sundar G, Gopal L, Khetan V (2019). Retinoblastoma in Asia. Eye (Lond).

[9] Jo DH, Lee K, Kim JH, Jun HO, Kim Y, Cho YL (2017). L1 increases adhesion-mediated proliferation and chemoresistance of retinoblastoma. Oncotarget..

[10] Benekli M, Smiley SL, Younis T, Czuczman MS, Hernandez-Ilizaliturri F, Bambach B (2008). Intensive conditioning regimen of etoposide (VP-16), cyclophosphamide and carmustine (VCB) followed by autologous hematopoietic stem cell transplantation for relapsed and refractory Hodgkin's lymphoma. Bone Marrow Transplant.

[11] Shirasawa M, Nakahara Y, Niwa H, Harada S, Ozawa T, Kusuhara S (2016). Interstitial pneumonia following administration of pegfilgrastim during carboplatin and etoposide chemotherapy for small-cell lung cancer. Mol Clin Oncol.

[12] Nagano H, Tachibana Y, Kawakami M, Ueno M, Morita Y, Muraoka M (2016). Patients with advanced ovarian Cancer administered Oral Etoposide following Taxane as maintenance chemotherapy. Case Rep Oncol.

[13] Benson DM, Fau EP, Lin TS, Fau LT, Blum W, Fau BW, Penza S, Fau PS, Avalos B, Fau AB, Copelan E (2007). High-dose melphalan versus busulfan, cyclophosphamide, and etoposide as preparative regimens for autologous stem cell transplantation in patients with multiple myeloma. Leuk Res.

[14] Ashley DM, Fau ML, Kerby T, Fau KT, Zalduondo FM, Fau ZF, Friedman HS, Fau FH, Gajjar A, Fau GA, Kun L (1996). Response of recurrent medulloblastoma to low-dose oral etoposide. J Clin Oncol.

[15] Song WP, Zhang CY, Zhang Y, Li Y, Cao R, Ye C (2017). Generation of etoposide-resistant subline of human retinoblastoma Y79 cells and preliminary study on the mechanism of drug resistance. Chin Med Biotechnol.

[16] Kim D, Pertea G, Trapnell C, Pimentel H, Kelley R, Salzberg SL (2013). TopHat2: accurate alignment of transcriptomes in the presence of insertions, deletions and gene fusions. Genome Biol.

[17] Storey JD, Tibshirani R (2003). Statistical significance for genomewide studies. Proc Natl Acad Sci U S A.

[18] Young MD, Fau WM, Smyth GK, Fau SG, Oshlack A, Oshlack A (2010). Gene ontology analysis for RNA-seq: accounting for selection bias. Genome Biol.

[19] Kanehisa M, Fau AM, Goto S, Fau GS, Hattori M, Fau HM, Hirakawa M, Fau HM, Itoh M, Fau IM, Katayama T (2008). KEGG for linking genomes to life and the environment. Nucleic Acids Res.

[20] Mao X, Fau CT, Olyarchuk JG, Fau OJ, Wei L, Wei L (2005). Automated genome annotation and pathway identification using the KEGG Orthology (KO) as a controlled vocabulary. Bioinformatics.

[21] Livak KJ, Schmittgen TD (2001). Analysis of relative gene expression data using real-time quantitative PCR and the 2(−Delta Delta C(T)) method. Methods..

[22] Snow K, Judd W (1991). Characterisation of adriamycin- and amsacrine-resistant human leukaemic T cell lines. Br J Cancer.

[23] McGranahan N, Swanton C (2015). Biological and therapeutic impact of intratumor heterogeneity in cancer evolution. Cancer Cell.

[24] Cui Q, Wang JQ, Assaraf YG, Ren L, Gupta P, Wei L (2018). Modulating ROS to overcome multidrug resistance in cancer. Drug Resist Updat.

[25] Lorendeau D, Dury L, Nasr R, Boumendjel A, Teodori E, Gutschow M (2017). MRP1-dependent collateral sensitivity of multidrug-resistant Cancer cells: identifying selective modulators inducing cellular glutathione depletion. Curr Med Chem.

[26] Ranjbar S, Khonkarn R, Moreno A, Baubichon-Cortay H, Miri R, Khoshneviszadeh M (2019). 5-Oxo-hexahydroquinoline derivatives as modulators of P-gp, MRP1 and BCRP transporters to overcome multidrug resistance in cancer cells. Toxicol Appl Pharmacol.

[27] Garcia-Mayea Y, Mir C, Masson F, Paciucci R, Me LL. Insights into new mechanisms and models of cancer stem cell multidrug resistance. Semin Cancer Biol. 2019. 10.1016/j.semcancer.2019.07.022 31369817

[28] Van Allen EM, Wagle N, Sucker A, Treacy DG, Johannessen CM, Goetz EM (2014). The genetic landscape of clinical resistance to RAF inhibition in metastatic melanoma. Cancer Discov.

[29] Shen H, Liang Z, Zheng S, Li X (2017). Pathway and network-based analysis of genome-wide association studies and RT-PCR validation in polycystic ovary syndrome. Int J Mol Med.

[30] Furukawa Y, Kawasoe T, Daigo Y, Nishiwaki T, Ishiguro H, Takahashi M (2001). Isolation of a novel human gene, ARHGAP9, encoding a rho-GTPase activating protein. Biochem Biophys Res Commun.

[31] Tcherkezian J, Lamarche-Vane N (2007). Current knowledge of the large RhoGAP family of proteins. Biol Cell.

[32] Takefuji M, Asano H, Mori K, Amano M, Kato K, Watanabe T (2010). Mutation of ARHGAP9 in patients with coronary spastic angina. J Hum Genet.

[33] Zhang H, Tang QF, Sun MY, Zhang CY, Zhu JY, Shen YL (2018). ARHGAP9 suppresses the migration and invasion of hepatocellular carcinoma cells through up-regulating FOXJ2/E-cadherin. Cell Death Dis.

[34] Wang T, Ha M (2018). Silencing ARHGAP9 correlates with the risk of breast cancer and inhibits the proliferation, migration, and invasion of breast cancer. J Cell Biochem.

[35] Ang BK, Lim CY, Koh SS, Sivakumar N, Taib S, Lim KB (2007). ArhGAP9, a novel MAP kinase docking protein, inhibits Erk and p38 activation through WW domain binding. J Mol Signal.

[36] Sun LJ, Zhang YP, Lou J (2017). ARHGAP9 siRNA inhibits gastric cancer cell proliferation and EMT via inactivating Akt, p38 signaling and inhibiting MMP2 and MMP9. Int J Clin Exp Pathol.

[37] Chen WX, Lou M, Cheng L, Qian Q, Xu LY, Sun L (2019). Bioinformatics analysis of potential therapeutic targets among ARHGAP genes in breast cancer. Oncol Lett.

[38] Song Y, Pan Y, Liu J (2019). The relevance between the immune response-related gene module and clinical traits in head and neck squamous cell carcinoma. Cancer Manag Res.

[39] Wang Y, Huang D, Shi J, Ma J, Zhao J, Li B (2014). Clinical treatment and prognostic observation for different pathological infiltrations in 537 patients with unilateral retinoblastoma. Chin Med J (Engl).

[40] Luo X, Ye HJ, Ding YG, Du Y, Yang HS (2014). Analysis of histopathology and prognosis of retinoblastoma after enucleation (in Chinese). Recent Adv Ophthalmol.

[41] Liu WL, Wu ZY, Yang HS, Yan JH (2010). Prognosis of postenucleation adjuvant therapy in retinoblastoma (in Chinese). Chin J Ocular Trauma Occup Eye Dis.

[42] Wang W, Li X, Lee M, Jun S, Aziz KE, Feng L (2015). FOXKs promote Wnt/beta-catenin signaling by translocating DVL into the nucleus. Dev Cell.

